# GLUT1 expression in pediatric adrenocortical tumors: a promising candidate to predict clinical behavior

**DOI:** 10.18632/oncotarget.19135

**Published:** 2017-07-10

**Authors:** Céline Pinheiro, Sara Granja, Adhemar Longatto-Filho, André M. Faria, Maria C.B.V. Fragoso, Silvana M. Lovisolo, Murilo Bonatelli, Ricardo F.A. Costa, Antonio M. Lerário, Madson Q. Almeida, Fátima Baltazar, Maria C.N. Zerbini

**Affiliations:** ^1^ Life and Health Sciences Research Institute (ICVS), School of Medicine, University of Minho, Braga, Portugal; ^2^ ICVS/3B's-PT Government Associate Laboratory, Braga/Guimarães, Portugal; ^3^ Barretos School of Health Sciences Dr. Paulo Prata – FACISB, São Paulo, Brazil; ^4^ Molecular Oncology Research Center, Barretos Cancer Hospital, São Paulo, Brazil; ^5^ Laboratory of Medical Investigation (LIM-14), School of Medicina, University of São Paulo, São Paulo, Brazil; ^6^ Unidade de Suprarrenal, Disciplina de Endocrinologia e Metabologia, Laboratório de Hormônios e Genética Molecular LIM42, Hospital das Clínicas, Faculdade de Medicina da Universidade de São Paulo, São Paulo, Brazil; ^7^ Instituto do Câncer do Estado de São Paulo - ICESP, Hospital das Clínicas, Faculdade de Medicina da Universidade de São Paulo, São Paulo, Brazil; ^8^ Hospital Universitário, Faculdade de Medicina da Universidade de São Paulo, São Paulo, Brazil; ^9^ Department of Internal Medicine, Division of Metabolism, Endocrinology and Diabetes, University of Michigan, Ann Arbor, MI, USA; ^10^ Departamento de Patologia, Faculdade de Medicina da Universidade de São Paulo, São Paulo, Brazil

**Keywords:** pediatric adrenocortical tumors, metabolic reprogramming, monocarboxylate transporter, glucose transporter, Warburg effect

## Abstract

**Background:**

Discrimination between benign and malignant tumors is a challenging process in pediatric adrenocortical tumors. New insights in the metabolic profile of pediatric adrenocortical tumors may contribute to this distinction, predict prognosis, as well as identify new molecular targets for therapy. The aim of this work is to characterize the expression of the metabolism-related proteins MCT1, MCT2, MCT4, CD147, CD44, GLUT1 and CAIX in a series of pediatric adrenocortical tumors.

**Methods:**

A total of 50 pediatric patients presenting adrenocortical tumors, including 41 clinically benign and 9 clinically malignant tumors, were included. Protein expression was evaluated using immunohistochemistry in samples arranged in tissue microarrays.

**Results:**

The immunohistochemical analysis showed a significant increase in plasma membrane expression of GLUT1 in malignant lesions, when compared to benign lesions (*p*=0.004), being the expression of this protein associated with shorter overall and disease-free survival (*p*=0.004 and *p*=0.001, respectively). Although significant differences were not observed for proteins other than GLUT1, MCT1, MCT4 and CD147 were highly expressed in pediatric adrenocortical neoplasias (around 90%).

**Conclusion:**

GLUT1 expression was differentially expressed in pediatric adrenocortical tumors, with higher expression in clinically malignant tumors, and associated with shorter survival, suggesting a metabolic remodeling towards a hyperglycolytic phenotype in this malignancy.

## INTRODUCTION

Adrenocortical tumors (ACTs) are common neoplasms with a prevalence higher than 3% in individuals older than 50 years, being the great majority of these tumors benign [1]. In opposition, adrenocortical carcinoma is a very rare and aggressive event, with an estimated annual worldwide incidence of 0.7-2 new cases per million [2–4]. In children, ACTs are an even more rare event, with an estimated annual worldwide incidence of 0.2-0.3 cases per million children younger than 15 years. Different groups have shown pediatric ACTs are different from adult ACTs [5–7], and, importantly, Weiss’ score [8], greatly useful in adults, cannot be applied to children to predict clinical behavior [6, 9, 10]. In fact, children with ACTs apparently with poor prognosis based on adult criteria often have a good clinical outcome [6, 11]. As a result, in 2003, Wieneke and collaborators, studying a series of 88 pediatric patients (<20 years) with ACTs, proposed a new score for children, based on invasion on vena cava, gross and microscopic features [11]. Although one study validates the reliability of Wieneke's scoring system in predicting malignancy in pediatric ACTs [12], the series was small (only 13 patients) and this system did not gain a great acceptance in the literature yet. Interestingly, Southern Brazil shows an unusually high incidence of pediatric ACTs, with values of 3.4-4.2 per million [2, 4, 13], a fact attributed to the high prevalence of the germline mutation p.R337H in *TP53* [14–16]. Currently, complete resection is the best treatment option to achieve cure in this type of cancer [17, 18]; however, long-term survival in children is still limited, with 5-year survival rates greatly varying (virtually from 0% to 100%) as a consequence of different disease presentations [11, 17, 19–21]. Prognostic factors include age, mitotic rate, tumor weight, tumor size, tumor extension, margin status, and presence of metastasis [19, 22, 23].

The metabolic reprogramming of cancer cells towards aerobic glycolysis, one of the recently defined hallmarks of cancer [24], has gained renewed attention as a potential prognostic and therapeutic tool. As normal epithelial cells become hyperproliferative, they reach the oxygen diffusion limit, leading to a state of intermittent hypoxia. These hypoxic cells can either suffer cell death or suffer a metabolic adaptation to a glycolytic phenotype. In this metabolic reprogramming, also known as the Warburg effect, cancer cells, either in the presence or absence of oxygen, produce energy mainly through glycolysis, whereas pyruvate, instead of being used for oxidative phosphorylation in the mitochondria, is converted to lactate, in a much less energetically efficient process. As a result, cancer cells consume high amounts of glucose to obtain the necessary ATP for proliferation, and produce high amounts of lactate, which is expelled from the cell through specific transporters, contributing to extracellular acidification. To sustain the high glycolytic rates, many metabolism-related proteins are upregulated in cancer, mainly due to the activity of hypoxia inducible factor 1 alpha (HIF-1α), the transcription factor involved in cellular responses to hypoxia [25, 26]. In this context, monocarboxylate transporters (MCTs), responsible for lactate transport, their chaperones CD147 and CD44, as well as the glucose transporter 1 (GLUT1) and the pH regulator carbonic anhydrase 9 (CAIX) arise as key players in the metabolic reprogramming of cancer cells [27–29].

In a previous study by our group, this metabolic reprogramming was described in adult adrenocortical carcinoma, which was associated with cancer cell aggressiveness [30]. Considering discrimination between benign and malignant tumors is more challenging in pediatric ACTs than in adult tumors [5], new insights in the metabolic profile of pediatric tumors may contribute to this distinction, to predict prognosis as well as to identify new molecular targets for directed therapy. As a result, the aim of the present work is to characterize the expression profile of the metabolism-related proteins MCT1, MCT2, MCT4, CD147, CD44, GLUT1 and CAIX in a series of pediatric ACTs, and its relation with clinical behavior.

## RESULTS

**Figure 1:**
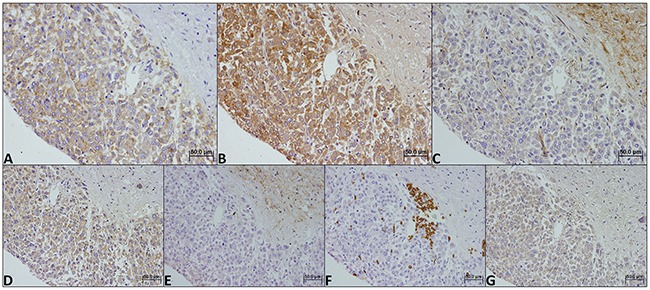
Immunohistochemical expression of MCT1 (A), MCT2 (B), MCT4 (C), CD147 (D), CD44 (E), GLUT1 (F) and CAIX (G) in a benign childhood adrenocortical tumor. Magnification: 400x

**Figure 2:**
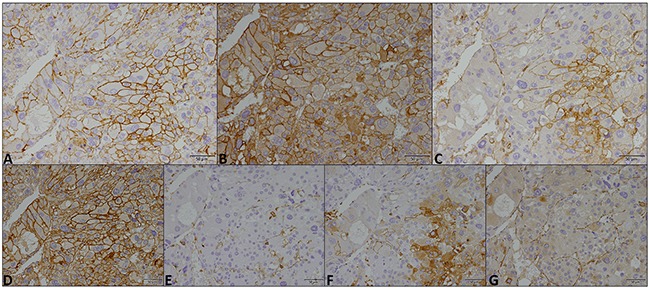
Immunohistochemical expression of MCT1 (A), MCT2 (B), MCT4 (C), CD147 (D), CD44 (E), GLUT1 (F) and CAIX (G) in a malignant childhood adrenocortical tumor. Magnification: 400x

**Figure 3:**
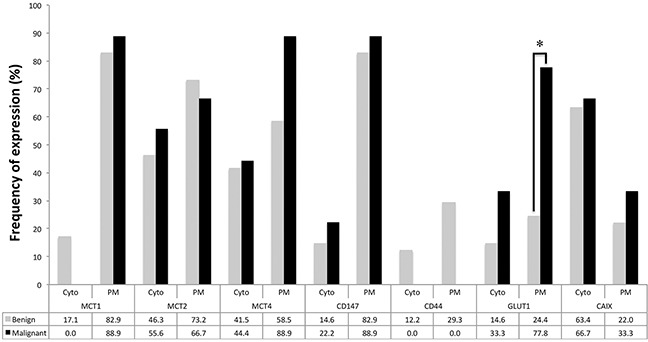
Frequency of expression of the different proteins analyzed in benign and malignant adrenocortical tumors Pearson's chi-square (χ^2^) test or Fisher's exact test was used to assess differences of expression frequency between benign and malignant tumors. Cyt - cytoplasmic expression; PM - plasma membrane expression; * *p*=0.004.

As shown in Figures 1, 2 and 3, the immuno-histochemical expression of the metabolism-related proteins in childhood ACTs was found in the cytoplasm, the plasma membrane or both localizations, with a predominance of plasma membrane expression (except for CAIX). All 3 MCT isoforms, as well as the chaperone CD147, were expressed at high frequency in the plasma membrane of most tumor samples (around 60-90%) and, for MCT4, the frequency of expression was higher in malignant than in benign tumors, however, with no statistical significance. Overall, CD44 was less frequently expressed in tumor samples, being absent from malignant tumors. Regarding the other metabolism-related proteins, GLUT1 and CAIX, a significant increase in the plasma membrane expression of GLUT1 was observed in malignant tumors, compared to benign tumors (*p*=0.004), but no differences in CAIX expression were observed. Evaluation of co-expression of MCTs with the other proteins at the plasma membrane (Table 1) showed a significant association between MCT4 and both GLUT1 and CAIX (*p*=0.002 and *p*=0.036, respectively).

**Table 1:** Co-expression of MCTs with CD147, CD44, GLUT1 and CAIX, in childhood adrenocortical tumor samples (benign and malignant). Only plasma membrane expressions were considered

	n	MCT1		MCT2		MCT4	
		Positive (%)	*p*	Positive (%)	*p*	Positive (%)	*p*
**CD147**			1.000		0.197		0.118
Negative	**8**	7 (87.5)		4 (50.0)		3 (37.5)	
Positive	**42**	35 (83.3)		32 (76.2)		29 (69.0)	
**CD44**			1.000		0.278		1.000
Negative	**38**	32 (84.2)		29 (76.3)		24 (63.2)	
Positive	**12**	10 (83.3)		7 (58.3)		8 (66.7)	
**GLUT1**			1.000		0.511		**0.002**
Negative	**33**	28 (84.8)		25 (75.8)		16 (48.5)	
Positive	**17**	14 (82.4)		11 (64.7)		16 (94.1)	
**CAIX**			0.173		0.140		**0.036**
Negative	**38**	30 (78.9)		25 (65.8)		21 (55.3)	
Positive	**12**	12 (100.0)		11 (91.7)		11 (91.7)	

**Figure 4:**
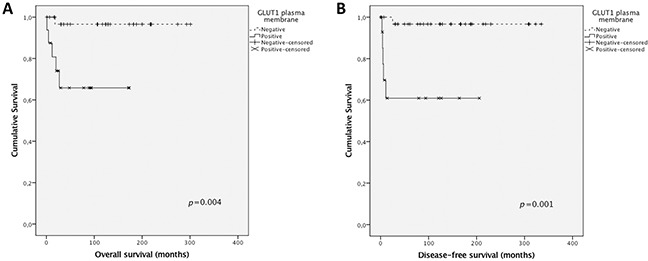
Overall and disease-free survival curves of pediatric adrenocortical carcinomas’ patients The results are stratified according to protein immunohistochemical expression. Only significant results are shown. Continuous line refers to positive expression while interrupted line refers to negative expression. **(A)** Association of GLUT1 plasma membrane expression with overall survival; **(B)** Association of GLUT1 plasma membrane expression with disease-free survival.

Taking into account the results obtained for GLUT1, the value of GLUT1 expression as a diagnostic marker was investigated. The positive predictive value of GLUT1 to characterize an adrenal mass as malignant was 41.2% while the corresponding negative predictive value to rule out malignancy was 93.9%, with a sensitivity of 77.8%, a specificity of 75.6% and an accuracy of 76.0%. Also, survival analysis showed a significant association of GLUT1 plasma membrane expression with shorter overall and disease-free survival (*p*=0.004 and *p*=0.001, respectively, Figure 4).

## DISCUSSION

In a previous study evaluating the expression and clinical significance of metabolism-related proteins in adult ACTs, MCT4, GLUT1 and CAIX plasma membrane expressions were shown to be significantly increased in carcinomas, when compared to adenomas. Moreover, MCT1, GLUT1 and CAIX plasma membrane expressions in carcinomas were significantly associated with poor prognosis, while MCT2 at the plasma membrane was associated with good prognosis [30]. In fact, although the majority of studies in ACTs using ^18^F-fluorodeoxyglucose positron emission tomography (^18^F-FDG-PET) rely on data obtained mainly or entirely from adult samples, evidence suggest a possible clinical relevance of the glycolytic metabolism, as ^18^F-FDG-PET may have both a diagnostic and prognostic value in adrenocortical malignancies [31–36]. Specifically in pediatric tumors, one study showed this technique was a satisfactory tool for metastasis screening [37] while another showed avid uptake of ^18^F-FDG by pediatric adrenocortical carcinoma [38]; however, both studies were based only on results from one patient with this type of cancer. Additionally, comparative studies in adult ACTs showed carcinomas, in opposition to adenomas, present biochemical markers indicative of increased glycolysis (such as lactate) [39], as well as increased levels of GLUT1 [40] and aldolase A, also a glycolytic enzyme, and decreased levels of proteins related to mitochondrial activity [41], also suggesting a metabolic reprogramming compatible with the Warburg effect. However, no similar studies are available for pediatric ACTs.

Following this line of evidence, in the present study, we characterized the expression of the glycolytic-related proteins: MCT1, MCT2, MCT4, CD147, CD44, GLUT1 and CAIX, in a series of pediatric ACTs. To the best of our knowledge, this is the first study aiming to characterize the metabolic profile of pediatric ACTs.

Herein, in line with the metabolic shift from an oxidative to a glycolytic phenotype, GLUT1 plasma membrane expression was significantly increased in malignant tissues, compared to benign tissues, similarly to results from adult adrenocortical carcinomas [30, 40]. Also, GLUT1 expression was associated with shorter overall and disease-free survival, indicating this protein can contribute to predict malignancy in pediatric ACTs, as, presently, there is no adequate scoring system to predict clinical behavior in this type of tumor [6, 9–11]. In opposition, although expressed at high frequencies at the plasma membrane of neoplastic cells, the 3 MCT isoforms as well as the chaperone CD147 were not significantly differently expressed in benign and malignant samples. In fact, with the exception of MCT4, expression frequencies of these proteins were very similar between groups. To note, the malignant tumor group included only 9 samples, which may explain the lack of significance in MCT4 plasma membrane expression frequencies between groups, as the statistical power was only sufficient to detect high differences in expression frequencies, such as the one found for GLUT1. The lack of CD44 in malignant samples suggests this protein is not a chaperone for MCT1 or MCT4 in malignant ACTs and has no role in pediatric adrenocortical malignancy. Finally, CAIX, a pH regulator upregulated in a variety of cancers, including adult adrenocortical carcinomas [30], showed similar expression between benign and malignant samples, suggesting other pH regulator, such as CAXII, should be involved in the acid-resistant phenotype associated with the hyperglycolytic phenotype depicted by GLUT1 overexpression. Interestingly, although MCT4 and CAIX were not differently expressed in malignant samples, compared to benign samples, MCT4 was associated with GLUT1 and CAIX, which is coherent with a protein profile remodeling towards a hyperglycolytic and acid-resistant phenotype.

Considering pediatric ACTs and adult ACTs show different genetic backgrounds, such as *TP53* mutations, as well as different molecular mechanism involved in tumorigenesis [5, 7], and oncogenes and tumor suppressors are important regulators of cancer cell metabolism [42], it is not surprising the expression of metabolism-related proteins in pediatric ACTs found in the present study shows some differences, when compared with a previous study with adult ACTs [30]. However, overall, the findings point at the same direction, which is a metabolic reprogramming towards a glycolytic phenotype in adrenocortical malignant tumors. Previous studies focusing on the biological aspects behind malignancy in pediatric ACTs have identified the insulin-like growth factor (IGF) system, specifically the pair IGF2/IGF1 receptor (IGF1R), as one of the molecular mechanisms involved in the process of tumorigenesis [43–46]. Although some of the signaling proteins downstream of IGF1R, such as Akt, mTOR and AMP-activated protein kinase (AMPK), have a pivotal role in cancer cell metabolic reprogramming [42, 47, 48], the possible role of IGF1R as a mediator of the Warburg effect has not been clearly established. Nevertheless, the use of a specific antibody targeting IGF1R in neuroblastoma and breast cancer xenograft models showed an antitumor activity, associated with a decrease in glucose uptake [49]. Additionally, IGF1R knockdown inhibited anchorage-independent growth of glioma cells, associated with a decreased glycolytic phenotype [50]. As a result, IGFR1 overexpression in pediatric malignant ACTs may be involved in a metabolic reprogramming towards a hyperglycolytic phenotype.

Targeting the metabolic reprogramming of cancer cells has been pointed out as a potential therapeutic tool and GLUT1 emerges as one of the promising target protein [27, 51, 52]. In fact, promising results on GLUT1 targeting, alone or in combination with conventional treatment, in a variety of tumor models are being described [53–55] and, based on the results from the present study, we anticipate pediatric adrenocortical carcinomas may also benefit from this therapeutic strategy.

Although showing promising results, the present investigation presents limitations, being the most critical the low number of cases available to be tested. Further studies, with more robust series, are warranted to increase statistical power and allow additional findings, including verification of MCT4 overexpression in malignant samples as well as association of the metabolic profile with clinicopathological data. Like in adult adrenocortical tumors, where GLUT1 was described as a stage-independent predictor of clinical outcome [40], the proteins herein analyzed may show clinicopathological significance.

In the present study, GLUT1 expression was differentially expressed in pediatric ACTs, with a higher expression in clinically malignant tumors than clinically benign tumors, and was associated with shorter overall and disease-free survival, suggesting a metabolic remodeling towards malignancy in these tumors.

## MATERIAL AND METHODS

### Human childhood adrenocortical tumor samples

The tumor series included 50 formalin-fixed paraffin-embedded ACTs (41 clinically benign and 9 clinically malignant lesions), retrieved from the files of the Pathology Department of the Clinical Hospital, School of Medicine, University of São Paulo, Brazil. Samples were organized into tissue microarrays (TMA) and each case was represented by three cores (1.0 mm diameter). Control samples (kidney) were included for TMA orientation. Cases were classified as clinically malignant if metastatic disease was detected. Clinicopathological data for the ACTs included age at diagnosis (non-normal distribution, median 2.2 years, range: 0.5 to 13 years), gender, *TP53 status*, tumor size (non-normal distribution, median 5.0 cm, range: 1.5 to 21.0 cm) and weight (non-normal distribution, median 40.0 mg, range: 4.0 to 650.0 mg), Weiss score and its individual histological parameters [8], staging (according to ENSAT system [56]), metastasis, progression-free survival and overall survival. Detailed information of the clinicopathological data for the childhood ACTs is presented in Table 2. The study was approved by the Local Ethic Committee (number 11090).

**Table 2:** Clinicopathological data of the pediatric adrenocortical tumor patients

Variable	n	%
**Age^#^ (n=50)**		
≥ 2.2 years	**28**	56.0
< 2.2 years	**22**	44.0
**Gender (n=50)**		
Female	**30**	60.0
Male	**20**	40.0
**TP53 germline mutation status (n=34)**		
Wild type	**8**	23.5
Mutated	**26**	76.5
**Tumor size (n=50)**		
< 5 cm	**20**	40.0
≥ 5 cm	**30**	60.0
**Tumor weight^#^ (n=48)**		
< 40.0 mg	**21**	43.8
≥ 40.0 mg	**27**	56.3
**Weiss score (n=49)**		
1	**4**	8.2
2	**5**	10.2
3	**5**	10.2
4	**7**	14.3
5	**11**	22.4
6	**9**	18.4
7	**6**	12.2
8	**1**	2.0
9	**1**	2.0
Nuclear grade* (n=18)		
Low	**4**	22.2
High	**14**	77.8
Mitotic index* (n=18)		
Low	**9**	50.0
High	**9**	50.0
**Atypical mitosis (n=18)**		
Absent	**8**	44.4
Present	**10**	55.6
**Necrosis (n=18)**		
Absent	**11**	61.1
Present	**7**	38.9
**Venous invasion (n=18)**		
Absent	**14**	77.8
Present	**4**	22.2
**Sinus invasion (n=18)**		
Absent	**16**	88.0
Present	**2**	11.1
**Capsular invasion (n=18)**		
Absent	**15**	83.3
Present	**3**	16.7
**Metastasis (n=50)**		
Absent	**41**	82.0
Present	**9**	18.0
**Staging (n=50)**		
I	**26**	52.0
II	**20**	40.0
III	**2**	4.0
IV	**2**	4.0

# Median values were used for cut-off as these variables followed a non-normal distribution. * Nuclear grade and mitotic index were defined according to Weiss score definitions [8].

### Immunohistochemistry

MCT1 immunohistochemistry was performed according to the avidin-biotin-peroxidase complex method (R.T.U. VECTASTAIN Elite ABC Kit (Universal), Vector Laboratories, Burlingame, CA), as previously described [57]. Immunohistochemistry for MCT2, MCT4, GLUT1, CD44 and CAIX was performed according to the streptavidin-biotin-peroxidase complex method (UltraVision Detection System Anti-polyvalent, HRP, Lab Vision Corporation, Fremont, CA), as previously described [58–60]. CD147 immunostaining was performed using a polymer system (UltraVision ONE Detection System: HRP Polymer Lab Vision Corporation, Fremont, CA) as previously described [61]. Negative controls were performed by the use of appropriate serum controls for the primary antibodies (N1698 and N1699, Dako, Carpinteria, CA). Colon carcinoma tissue was used as positive control for MCT1, MCT4, CD147 and CD44, head and neck squamous cell carcinoma was used for GLUT1, and normal stomach was used for CAIX. Tissue sections were counterstained with hematoxylin and permanently mounted. Detailed aspects for each antibody used are depicted in Table 3.

**Table 3:** Detailed aspects for each antibody used in immunohistochemistry

Protein	Antigen retrieval	Antibody (product # and company)	Antibody dilution andincubation time
**MCT1**	Citrate buffer (0.01 M, pH=6), 98°C, 20′	AB3538P Merck-Millipore	1:200, overnight
**MCT2**	Citrate buffer (0.01 M, pH=6), 98°C, 20	sc-50322 Santa Cruz Biotechnology	1:200, 2 hours
**MCT4**	Citrate buffer (0.01 M, pH=6), 98°C, 20′	sc-50329 Santa Cruz Biotechnology	1:500, 2 hours
**CD147**	EDTA (1 mM, pH=8), 98°C, 20′	sc-71038 Santa Cruz Biotechnology	1:400, overnight
**CD44**	Citrate buffer (0.01 M, pH=6), 98°C, 20	MCA2726 AbD Serotec	1:2000, 2 hours
**GLUT1**	Citrate buffer (0.01 M, pH=6), 98°C, 20′	ab15309-500 AbCam	1:500, 2 hours
**CAIX**	Citrate buffer (0.01 M, pH=6), 98°C, 20′	ab15086 AbCam	1:2000, 2 hours

### Immunohistochemical evaluation

Sections were scored semi-quantitatively for extension of expression in cancer cells as follows: 0: no immunoreactive cells; 1: <5% of immunoreactive cells; 2: 5-50% of immunoreactive cells; and 3: >50% of immunoreactive cells. Also, intensity of staining was scored semi-qualitatively as follows: 0: negative; 1: weak; 2: intermediate; and 3: strong. The final score was defined as the sum of both parameters (extension and intensity), and grouped as negative (score 0 and 2) and positive (score 3-6), as previously described [57]. Protein expression in the different cellular localizations (cytoplasm and plasma membrane) was evaluated separately.

### Statistical analysis

Data were stored and analyzed using the IBM SPSS Statistics software (version 23, IBM Company, Armonk, NY). All comparisons were examined for statistical significance using Pearson's chi-square (χ^2^) test or Fisher's exact test (depending on group size). Overall survival was defined as the time from the date of complete tumor resection to death related to adrenocortical cancer or last follow-up. Disease-free survival was defined as the time from the date of complete tumor resection to the first radiological evidence of disease relapse or death. Overall and disease-free survival curves were estimated by the method of Kaplan-Meier and data compared using the log-rank test. Due to the low number of malignant lesions (9/50, 18%), no statistical analysis associating the expression of the metabolism-related proteins with the clinicopathological variables was performed. The threshold for significant *p* values was established as *p*<0.05.
